# Giant Urinary Bladder Diverticula presenting as Epigastric Mass and Dyspepsia

**DOI:** 10.5812/numonthly.18918

**Published:** 2014-07-05

**Authors:** Santosh Kumar, Kumar Jayant, Yogesh Barapatra, Jyotsana Rani, Swati Agrawal

**Affiliations:** 1 Department of Urology, Postgraduate Institute of Medical Education and Research, Chandigarh, India

**Keywords:** Giant Bladder Diverticulum, Benign Hyperplasia of Prostate, Open Diverticulectomy

## Abstract

**Introduction:**

Bladder diverticulum is a result of bladder mucosa and submucosa herniation through the muscularis propria of bladder wall. Bladder diverticula are mostly seen in the elderly men in association with benign prostatic hyperplasia (BPH).

**Case Presentation:**

A 74-year-old man presented with complaints of vague epigastric discomfort, dyspepsia, and mild lower urinary tract symptoms. An ultrasonography of the abdomen showed bilateral hydroureteronephrosis, large cystic lesion with the size of 26.3 × 20.5 cm and in continuation of urinary bladder and prostate of 70 mL volume. Voiding cystourethrogram revealed a large diverticulum with its neck communicating with bladder on posterior aspect. Abdominopelvic contrast-enhanced computed tomography revealed bilateral hydronephrosis with large bladder diverticulum of 27.3 × 21.5 cm in size with smooth diverticular wall. On cystoscopy, the neck of diverticulum was seen at the posterior wall of bladder. Open prostatectomy and diverticulectomy were done simultaneously (Figure 3). Postoperative course was uneventful. The histopathological assessment showed features of chronic inflammation without any evidence of malignancy. On the third postoperative day, the urethral catheter was removed and suprapubic catheter was clamped. Patient was voiding well and cystography done on day 12 revealed smooth bladder contour without any leakage; hence, suprapubic catheter was removed. Patient was discharged in satisfactory condition.

**Conclusions:**

The elderly men are at high risk of developing bladder diverticulum, which may be due to high prevalence BPH in this group. Although presentation of bladder diverticulum is nonspecific, its effect on renal system is significant. Therefore, awareness of patients and physicians is necessary to prevent its consequences.

## 1. Introduction

Bladder diverticulum is described as bladder mucosa and submucosa herniation through bladder muscular wall. The diverticulum maintain its connection with the bladder via a narrow neck. The function of urinary bladder is usually impaired, i.e. poor emptying due to absence of muscular layer in its wall. Diverticula are usually asymptomatic and are discovered incidentally during examination for other reasons. Here we reported a rare case of the giant urinary bladder diverticulum that presented with epigastric discomfort, dyspepsia, and mild features of lower urinary tract symptoms.

## 2. Case Report

**Figure 1.**
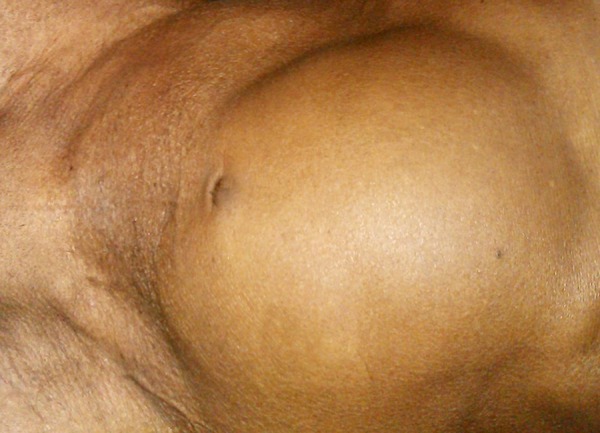
A Lump Extending From Epigastrium to Pubic Symphysis

**Figure 2.**
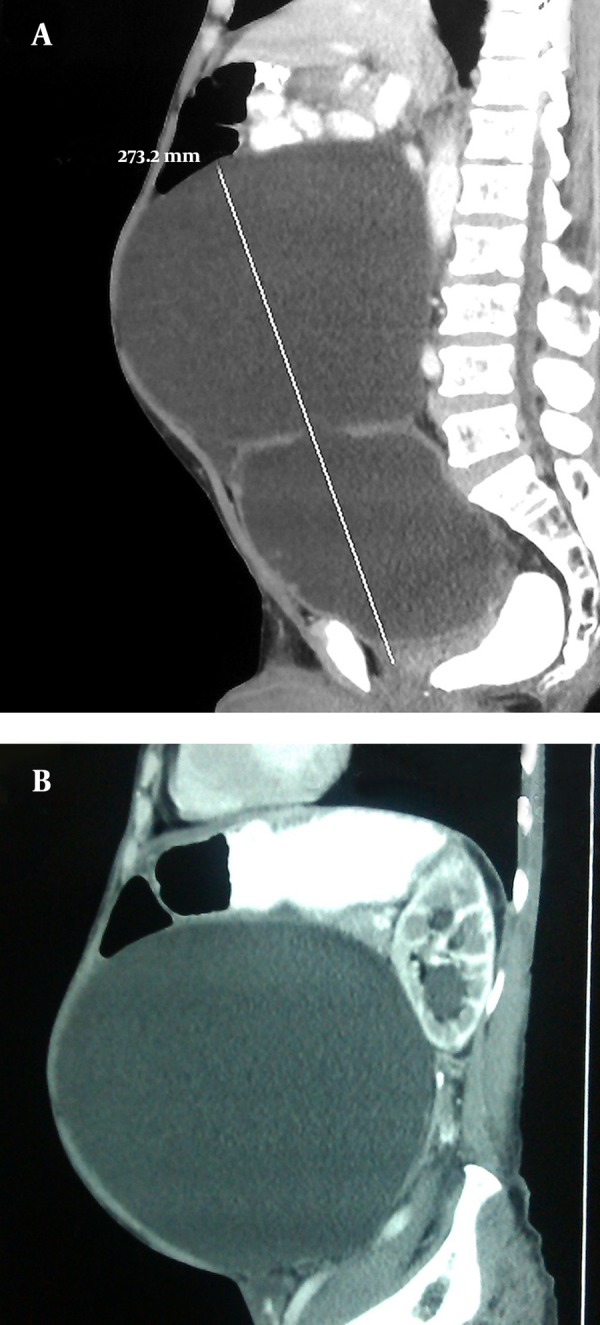
Contrast-Enhanced Computed Tomography of Abdomen Showing Giant Urinary Bladder Diverticulum

**Figure 3.**
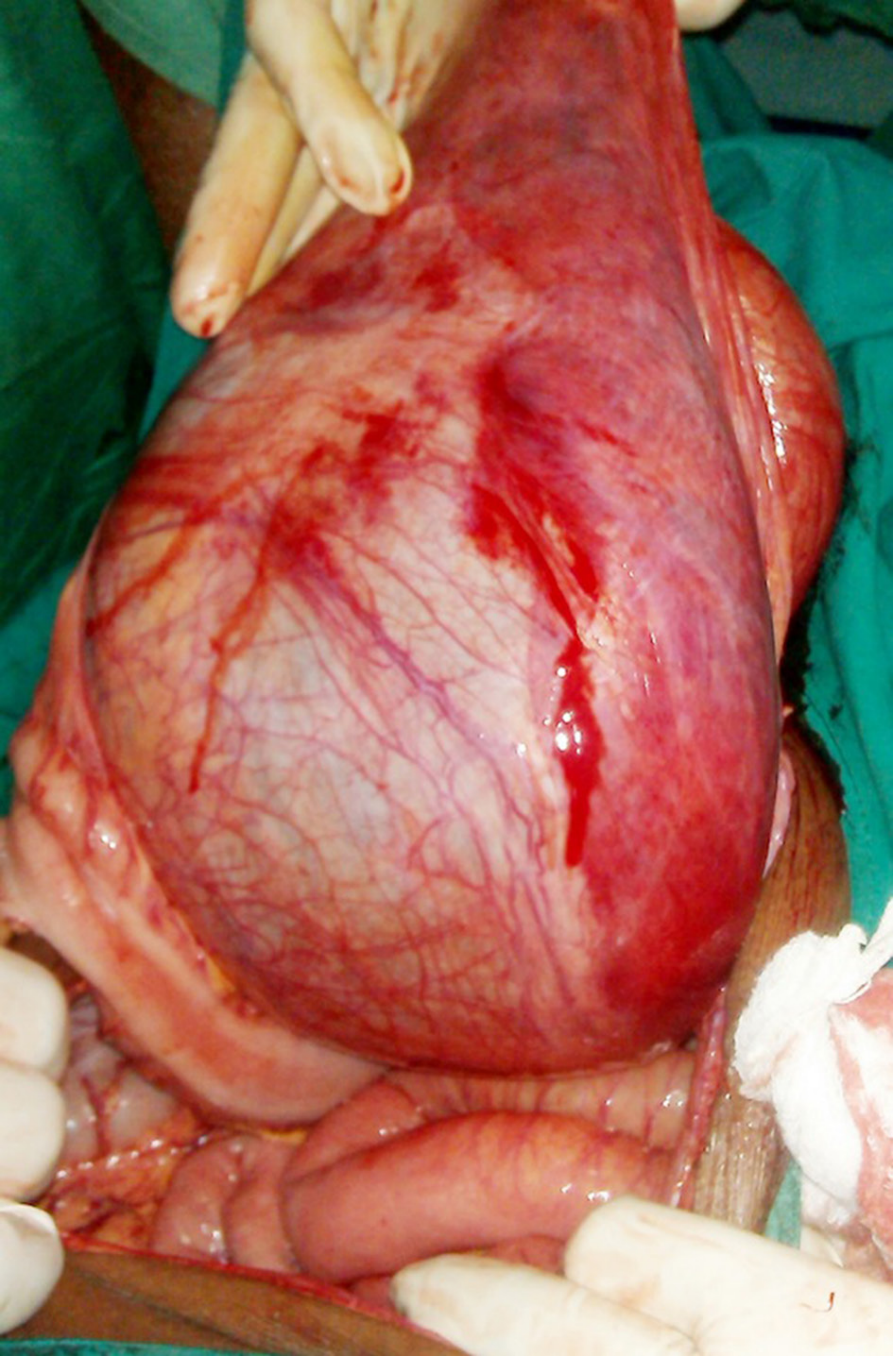
Intraoperative Photograph Showing the Giant Bladder Diverticulum

A 74-year-old man presented to the outpatient department of our institute with complaints of dyspepsia, fullness in the central abdomen extending from epigastrium to umbilicus, and mild lower urinary tract symptoms such as dysuria, increased urinary frequency, nocturia, and diminished urinary stream for the preceding two years (Figure 1). On physical examination, there was a palpable lump in the upper abdomen extending from the epigastrium to the pubic symphysis, which was nontender, firm in consistency with indistinct lower border. A urethral catheter was placed immediately and 2000 mL of urine was drained that led into improvement of abdominal distension and discomfort. His initial investigation revealed hemoglobin of 11 g/dL, TLC (Total Leukocyte Count) of 11000/µL, serum urea of 66 mg/dL, serum creatinine of 2.1 mg/dL, and serum PSA of 2.4 ng/mL. The ultrasonography of the abdomen showed bilateral hydroureteronephrosis and a large cystic lesion of 26.3 × 20.5 cm size in continuation of urinary bladder. Voiding cystourethrogram revealed a large diverticulum with its neck communicating with posterior wall of bladder. Abdominopelvic contrast-enhanced computed tomography revealed bilateral hydronephrosis with large bladder diverticulum with 27.3 × 21.5 cm size and smooth diverticular wall (Figure 2). Cystoscopy revealed diffuse trabeculation inside the urinary bladder. The neck of diverticulum was seen on the posterior wall of bladder. The inner aspect of diverticulum was smooth and biopsy showed features of inflammation without any evidence of malignancy. Open prostatectomy and diverticulectomy through a midline incision were done simultaneously (Figure 3). Postoperative course was uneventful. The histopathological assessment of the specimen revealed that the wall diverticulum was composed of mucosa and lamina propria with scattered thin muscle fibers with features of chronic inflammation without any evidence of malignancy. On day 12, follow-up cystography showed smooth bladder contour without leakage. Hence, the suprapubic catheter was removed. Uroflowmetry showed a peak flow rate of 11 mL/s on voiding 256 mL urine, with 15 mL residual urine and improved serum creatinine of 1.7 mg/dL and serum urea of 40 mg/dL. Patient was discharged in satisfactory condition and was doing well on follow-up.

## 3. Discussion

Mucosal outpouching through areas of congenital or acquired weakness in bladder wall leads to formation of diverticulum. Bladder diverticula are classified as follows:

1. congenital type, which is uncommon and is usually seen in association with posterior urethral valve or neurogenic bladder; and
2. acquired or pulsion type, which is usually seen secondary to bladder outlet obstruction.

The most common etiologies are benign hyperplasia of prostrate (BPH), urethral stricture, or voiding dysfunction.

Considering the high prevalence of BPH in the elderly men, many neglected patients are at risk of developing bladder diverticula and its occurrence is far higher than what is usually diagnosed. One of the largest study done by Shakeri et al. has reported the prevalence rate of 27.4% on cystoscopy and 40% on cystography (1). The main reason behind silent occurrence of these diverticula is absence of any characteristic clinical features suggestive of bladder diverticulum. Few patients present with features of lower urinary tract symptoms, occasional “instalment voiding”, or large amount of residual urine, which is often infected (2, 3). The most frequently reported complications are recurrent urinary tract infections (up to 68%), malignant intradiverticular tumors (2%-20%), vesicoureteral reflux or ureteral obstruction (5%-15%), and spontaneous rupture (4, 5). Our case was an excellent example of diverticulum development in presence of BPH with mild symptoms. To our knowledge, it had the largest size among ever-reported giant bladder diverticula, which presented as an epigastric mass.

Bladder diverticula may be suspected in any patient presenting with symptoms of recurrent infection, difficulty in voiding, or abdominal fullness that suggest blockage of the bladder outlet and urinary stasis. When suspected, the voiding cystourethrogram is an excellent means of detecting bladder diverticula as it gives information regarding site, size, and associated vesicoureteral reflux. In addition, diverticula must be evaluated by cystoscopy and biopsy, which not only aids in diagnosis but also helps in excluding any carcinomatous changes. The most common malignancy seen is transitional cell carcinoma (70%-80%) (6). The bladder functioning and presence of any obstruction in its outlet may be assessed with urodynamic studies; however, urodynamic studies might be fallacious in the presence of a large diverticulum. Abdominal ultrasonography and contrast-enhanced computed tomography must be consider in evaluating the extent of diverticulum and pressure changes in kidney secondary to obstruction. Treatment of bladder diverticulum is primarily directed towards the cause of its development than diverticulum per se; in cases of small diverticulum, relieving the obstruction leads into resolution of diverticulum (7); however, large diverticulum needs attention and open diverticulectomy is surgery of choice. Although laparoscopic and robotic-assisted methods are reported, open modality remains treatment of choice for large cases such as ours (8).

The elderly are at increased risk of developing giant bladder diverticulum. BPH is a gradually developing pathology; hence, people adapt to the disease process and tend to overlook the symptoms. Although presentation of bladder diverticulum may be nonspecific, it poses severe threat to the renal system. Thus, awareness of both patient and physician is necessary in order to early diagnosis of the condition. Which helps in implementation desired therapeutic intervention.
